# A comprehensive evaluation system for ultrasound-guided infusion of human umbilical cord-derived MSCs in liver cirrhosis patients

**DOI:** 10.1093/stcltm/szae081

**Published:** 2024-11-08

**Authors:** Guo Zhou, Yijuan You, Binghua Wang, Simin Wang, Tianhang Feng, Chunyou Lai, Guangming Xiang, Ke Yang, Yutong Yao

**Affiliations:** Department of Ultrasound, Sichuan Provincial People’s Hospital, University of Electronic Science and Technology of China, Chengdu 610072, People’s Republic of China; Department of Ultrasound, Wenjiang Hospital of Sichuan Provincial People’s Hospital, Chengdu 611100, People’s Republic of China; Department of Ultrasound, Wenjiang Hospital of Sichuan Provincial People’s Hospital, Chengdu 611100, People’s Republic of China; Department of Ultrasound, Wenjiang Hospital of Sichuan Provincial People’s Hospital, Chengdu 611100, People’s Republic of China; Department of Hepatobiliary and Pancreatic Surgery Center, Cell Transplantation Center, Sichuan Academy of Medical Sciences, Sichuan Provincial People’s Hospital, School of Medicine, University of Electronic Science and Technology of China, Chengdu 610072, People’s Republic of China; Department of Hepatobiliary and Pancreatic Surgery Center, Cell Transplantation Center, Sichuan Academy of Medical Sciences, Sichuan Provincial People’s Hospital, School of Medicine, University of Electronic Science and Technology of China, Chengdu 610072, People’s Republic of China; Department of Hepatobiliary and Pancreatic Surgery Center, Cell Transplantation Center, Sichuan Academy of Medical Sciences, Sichuan Provincial People’s Hospital, School of Medicine, University of Electronic Science and Technology of China, Chengdu 610072, People’s Republic of China; Department of Ultrasound, Sichuan Academy of Medical Sciences, Sichuan Provincial People’s Hospital, Chengdu 610072, People’s Republic of China; Department of Hepatobiliary and Pancreatic Surgery Center, Cell Transplantation Center, Sichuan Academy of Medical Sciences, Sichuan Provincial People’s Hospital, School of Medicine, University of Electronic Science and Technology of China, Chengdu 610072, People’s Republic of China

**Keywords:** liver cirrhosis, mesenchymal stem cells transplantation, pig-tail catheter

## Abstract

**Background:**

Infusion of mesenchymal stem cells (MSCs) via portal vein is one of the main ways for MSCs transplantation to treat liver cirrhosis (LC). As the tissue of LC showed diffuse fibrosis and thickened Glission sheath, the soft pig-tail catheter, or central venous catheter can not successfully insert the portal vein. Thus, our study used an improved method and performed a relatively comprehensive system to evaluate the effect for human umbilical cord-derived mesenchymal stem cells (hUC-MSCs) transplantation.

**Method:**

Fifteen patients with hepatitis B-related cirrhosis were enrolled in the study, and we performed hUC-MSCs transplantation via portal vein by using an 16-G needle and 0.035-inch guide wire combined with 7FR “retentional metal stiffner trocar” of pig-tail catheter under the guidance of contrast-enhanced ultrasound. Serum liver function, fibrotic indicators, tissue stiffness, coagulation function, and hemodynamics were measured at weeks 4, 12, and 24 after MSCs transplantation. Liver biopsy was performed before and 24 weeks after hUC-MSCs transplantation.

**Result:**

After hUC-MSCs transplantation, the prothrombin time was lower than before. The levels of hyaluronic acid and IV-C(Type IV collagen) in fibrotic indicators were significantly reduced, and the Young’s modulus was also decreased. Moreover, liver biopsy showed that the lytic necrosis of hepatocyte was decreased. In liver hemodynamics, the portal vein diameter was decreased after hUC-MSCs transplantation.

**Conclusion:**

hUC-MSCs transplantation can alleviate liver damage caused by LC. The improved “retentional metal stiffner trocar” of pig-tail catheter was safe and effective in the infusion of hUC-MSCs transplantation, which is worth promoting in clinical practice.

## Graphical abstract

**Figure:**
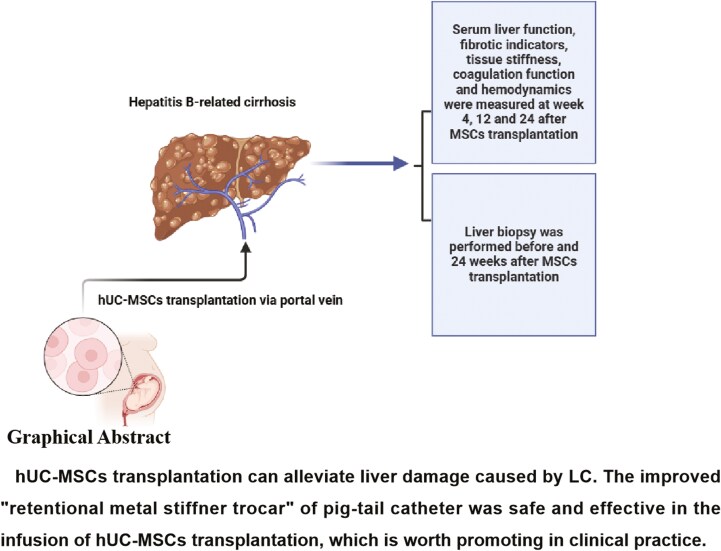

**Lessons learned**

1. There is no special treatment for patients with hepatitis B cirrhosis in the advance stage. In addition, the tissue of liver cirrhosis (LC) showed diffuse fibrosis and thickened Glission sheath, the soft pig-tail catheter, or central venous catheter can not always successfully insert the portal vein. Thus, our study used an 16-G needle and 0.035-inch guide wire combined with 7FR “retentional metal stiffner trocar” of pig-tail catheter to infuse the mesenchymal stem cells (MSCs) into the liver, and performed a relatively comprehensive system to evaluate the effect for MSCs transplantation.
2. In our study, 15 patients with hepatitis B cirrhosis were followed after MSCs transplantation. Indicators of liver function and cirrhosis were examined at 4, 12, and 24 weeks. In addition, liver biopsy was performed again at week 24 to assess the liver pathology.
3. Our results showed that MSCs transplantation can alleviate some liver damage caused by LC. The improved method was safe and effective in MSCs transplantation and helped to improve the successful rate. In addition, the relatively comprehensive system proposed in this study would help patients to be evaluated after transplantation. In conclusion, our results are worth promoting in clinical practice.

**Significance statement**

Infusion of mesenchymal stem cells (MSCs) via portal vein is one of the main ways for MSCs transplantation to treat liver cirrhosis (LC). As the tissue of LC showed diffuse fibrosis and thickened Glission sheath, the soft pig-tail catheter, or central venous catheter can not successfully insert the portal vein. Thus, our study used an improved method and performed a relatively comprehensive system to evaluate the effect for human umbilical cord-derived mesenchymal stem cells (hUC-MSCs) transplantation.

## Introduction

Hepatitis B virus (HBV) infection is one of the common infectious diseases in the world. In 2019, it was estimated that 296 million people were infected with HBV worldwide and nearly 1 million died.^1^ Liver cirrhosis (LC) is characterized with the degeneration and necrosis of hepatocytes, the formation of false lobules and regenerative nodules, and loss of liver function.^2^ LC is one of the major serious complications of HBV,^3^ with an estimated 331 000 people died from HBV-related cirrhosis in 2019.^4^ In addition, LC is also one of the main risk factors to develop liver cancer.^5^ The formation of reactive oxygen species is currently considered to be a major driver of LC,^6^ but the antioxidant therapy has limited effect.^5^ In fact, there is currently no effective drugs to prevent the progression of LC, which brought serious economic and medical burdens to society.

Although there are no drugs that can effectively treat LC, liver transplantation is the only way to treat end-stage liver disease. However, the practical problem is that it is difficult to get suitable donors. Moreover, among adult patients of LC, the mortality rate waiting for a liver transplant is close to 11%.^7^ Thus, it is urgent to find a new treatment to prevent LC from progressing.

Immune dysfunction is another major driver of LC, and regulating immune dysfunction is consider to be an effective way to prevent the progression of LC.^8^ Mesenchymal stem cells (MSCs) are a group of self-renewing cells capable of differentiating into a variety of cell types and playing a vital role in tissue healing and regeneration of damaged tissues.^9^ Due to the low immunogenicity^10^ and the ability to regulate the function of immune cells of MSCs,^11,12^ some studies showed that the autologous and allogeneic MSCs play a vital role in alleviating fibrosis and improving liver function.^13-16^ Thus, treatment for liver fibrosis by MSCs is safe, effective, and has a good research prospect.

Moreover, it is worth exploring how to transplant MSCs to the cirrhotic liver. The common administration of intrahepatic transplantation for MSCs include intravenous hepatic injection, arterial hepatic injection, and peripheral intravenous injection.^17^ The study of Barbash IM et al^18^ showed that the administration for MSCs transplantation via peripheral veins mainly accumulate in the lungs, while the liver, heart, and spleen are less distributed in the rat model of myocardial infarction. In addition, transplantation of MSCs via the hepatic artery usually requires X-ray guidance, but this method has a higher risk of radiation and bleeding. In contrast, the ultrasound-guided MSCs transplantation via the portal vein is more safe. In addition, the portal vein supplies 70%-80% of blood to liver, and the administration of MSCs via the portal vein can be accurately positioned and directly reach the liver blood sinuses. More importantly, multiple studies have shown that the MSCs transplantation via the portal vein is benefit over peripheral intravenous injection and can prevent the progression of LC.^19-21^ Thus, we should firstly choose portal vein for MSCs transplantation to LC.

However, the liver tissue of LC presents with diffuse fibrosis and the formation of regenerative nodules, resulting in rough surface of liver, increased hardness of liver parenchyma, and the disorders of intrahepatic vascular structure (such as portal vein spongiosis).^2^ In addition, the accumulation of inflammatory cells and matrix deposition around the portal vein lead to a thickening of Glission sheath.^22^ These pathological changes require us to select a more suitable catheter for MSCs transplantation. If a simple pig-tail catheter or central venous catheter is used, the portal vein cannot be successfully inserted from the rigid liver parenchyma and thickened Glission sheath by the guide wire due to the low rigidity of the hose material.^23^ Therefore, we need to select a suitable catheter for MSCs transplantation.

It is also important to evaluate the effects of MSCs transplantation in the treatment of LC, but there is lack of a comprehensive evaluation system for it. In recent years, the Two-dimensional shear-wave elastography ultrasound (TSWE)^24-26^ and contrast-enhanced ultrasound (CEU)^27-29^ have been used to effectively evaluate liver fibrosis. In addition, pathological changes play a vital important role in the evaluation of MSCs transplantation for the treatment of LC. Therefore, other than the traditional indicators related to liver fibrosis, the TSWE, CEU and the pathological changes were also comprehensively used to evaluate MSCs transplantation in the treatment of LC.

Above all, our study used a “retentional metal stiffner trocar” of pig-tail catheter for MSCs transplantation via portal vein to evaluate the safety and efficacy of this improved method in patients with LC and evaluate the effect of MSCs transplantation by a relatively comprehensive evaluation system.

## Methods

### Patients enrolled in the study

Fifteen patients with end-stage cirrhosis of hepatitis B at SiChuan Provincial People’s Hospital in China were enrolled in the study from November 2021 to December 2022. The clinical baseline data and the flow chart of this study are shown in supplemental material. Thirteen of the 15 patients were male and 2 were female. The mean age of them was 47.73 ± 7.08 years, the mean course of disease was 20.33 ± 9.75 years, the mean BMI was 23.79 ± 2.57 kg/m^2^, and the Child-Pugh grade of pathology was grade B in all of them. Among the 15 patients, HBV DNA quantification was 2.06 × 10^5^ IU/mL in 1 patient, and it was <1 × 10^3^ IU/mL in the other patients. In addition, all patients have received antiviral therapy.

### Ethical approval

All patients have signed the informed consent forms and received approval from the institute’s Committee on Human Research of SiChuan Provincial People’s Hospital (2019001). This study is registered with Chictr.org.cn, number ChiCTR2100052843.

### Inclusion and exclusion criteria

Inclusion criteria for LC patients were as follow:

(1) The age of men and/or women between 20 and 65; all patients had a definite diagnosis of chronic hepatitis B, and their HBV DNA measurement was outside the normal range prior to receiving anti-HBV drugs.
(2) All patients with decompensated cirrhosis of chronic hepatitis B was diagnosed with a disease course of more than 5 years;
(3) Criteria for decompensation of chronic hepatitis B cirrhosis: Child-Pugh grade B or Child-Pugh grade C, with reduced serum ALB, or more than 2-fold increase in bilirubin levels, or mild coagulation abnormalities, and no abdominal fluid accumulation or controlled abdominal fluid accumulation. Portal hypertension (moderate to severe lower esophageal fundus varicose veins, the number of platelet less than 1 × 10^5^/DL);
(4) Orthotopic liver transplantation is not clinically suitable, or there is no clear liver source;
(5) No severe bleeding tendency or active bleeding;
(6) There was no hepatic encephalopathy in clinical assessment;

Exclusion criteria for LC patients were as follow:

(1) Age < 20 years or > 65 years;
(2) Other causes of cirrhosis include alcoholic hepatitis, viral hepatitis C, and autoimmune hepatitis;
(3) Patients with liver malignancy and/or family history of liver malignancy in three immediate family members;
(4) The patient had a history of tumors in other organs;
(5) Prothrombin time is extended by more than 3 seconds;
(6) Human ALB was transfused within 3 weeks before enrollment;
(7) Clinically significant upper gastrointestinal hemorrhage occurred within the first 4 weeks of enrollment;
(8) The patient had spontaneous peritonitis;
(9) An active infection caused by a virus or bacteria;
(10) Pregnant or lactating women;
(11) Hypersensitivity to ultrasound contrast media, inability to cooperate with breathing, BMI≥30kg/m^2^;

### Evaluation of indicators related to liver fibrosis

The indicators of liver function, coagulation function and liver fibrosis in serum were analyzed by automatic biochemical analyzer.

### Color Doppler ultrasound examination

The cannon aplio i800 color Doppler diagnostic instrument was used in our study. The ultrasound probe was placed in the right intercostal scan, and the first hepatic portal and main portal vein were fully displayed. The distance between the inner wall of the portal vein and the blood flow spectrum of the portal vein were measured at the widest point in front of the inferior vena cava and perpendicular to the tube wall. The Angle between the sampling line and the blood flow was ≤60°, and the color sampling frame was taken to an appropriate size and the sampling volume was set to 2 mm. When the blood flow signal in the main portal vein was well filled, the patient was asked to temporarily hold his breath, and the portal vein blood flow spectrum was measured at the same time. The sampling position was fixed as much as possible during each measurement of the portal vein blood spectrum in the same patient, and the measurement was repeated three times each time, and the final result was averaged.

### Contrast-enhanced ultrasound

The cannon aplio i800 color Doppler diagnostic instrument was used with a convex array probe, the probe frequency range was about 3.5-5.0MHz, the mechanical index was set to 0.08, and the injection sulfur hexafluoride microbubble from Bracco Italy was used as the ultrasonic contrast agent.

#### Preparation method of contrast agent

The white dry powder of sulfur hexafluoride was dissolved with 5 mL 0.9% sodium chloride injection before use and shaken to make milky white suspension for reserve use. The ROI of tracking sampling during the imaging process was Ellipse 2D (Boundary BOX: 2.78 mm × 2.78 mm).

### Two-dimensional shear wave elastic ultrasound

As recommended by the guidelines,^30^ S5 or S6 segments of liver should be selected as far as possible each time. When the 2D B-mode image is clearly displayed, the sampling frame should be appropriately enlarged. The sampling frame should be placed in the center of the entire image, and its upper edge should be placed 1-2 cm below the liver capsule, with a maximum depth of no more than 5 cm, and interference from non-target structures such as large blood vessels and gallbladder in the liver should be avoided. The patient was require to breathe gently for 3-5s, freeze the elastic image after it reaches uniform stability, and measure the liver elasticity value, and the measurement ROI is fixed at 10 mm.

### Ultrasound-guided liver biopsy

After general anesthesia, the patient was positioned under ultrasound guidance and percutaneous liver biopsy was performed using Coaxial achieve 16GX15cm tissue biopsy needle. Three pieces of liver tissue were taken and fixed in 10% neutral formalin buffer. Slices were made according to standard methods. HE staining and Masson staining were performed, and inflammation and fibrosis stages were scored according to Ishak grading and staging system.^31^

### MSCs transplantation

#### Material

16-G needle, 0.035-inch guide wire and “retentional metal stiffner trocar” of pig-tail catheter (7FR × 30 cm). Human umbilical cord mesenchymal stem cell were obtained from Shanghai Quansheng Biotechnology Co., Ltd (QSC1012021070601-2).

**Figure 1.**
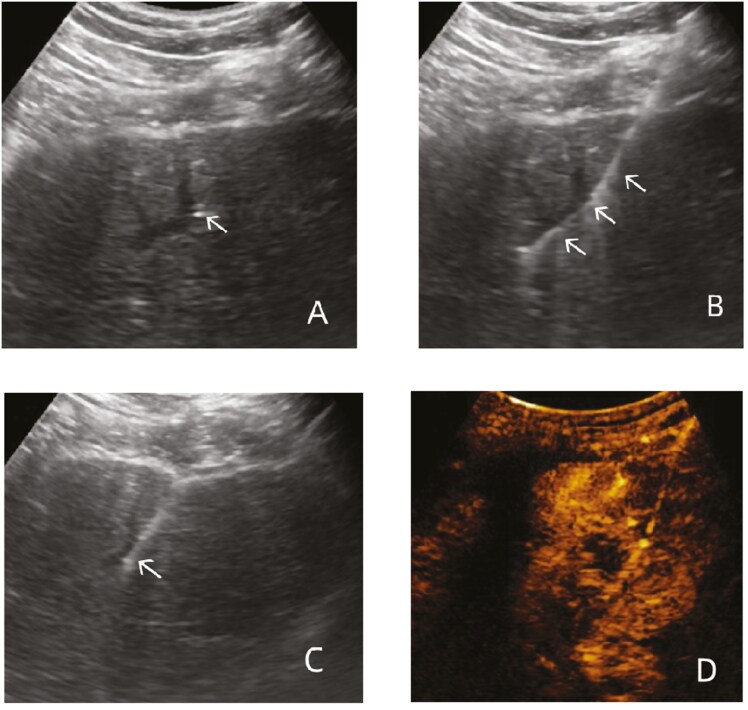
Ultrasound-mediated catheterization of the portal vein. (A) The 16G needle was inserted via the liver into the branch of the left lower external of the portal vein. The white arrow indicates that the tip of the needle is located on the left lower external of the portal vein; (B) The 0.035 inch guide wire was inserted along the needle core. The white arrow indicates the path of guide wire placement; (C) the “retentional metal stiffner trocar” of pig-tail catheter breaks through the capsule and parenchyma of liver into the portal vein. The white arrow indicates the “retentional metal stiffner trocar” of pig-tail catheter; (D) the Sonovue was injected by a drainage tube. Contrast-enhanced ultrasound showed that the contrast agent distributed diffusely into the hepatic parenchyma along the portal vein of the liver.

After the liver biopsy was successful, percutaneous portal vein puncture catheter (“retentional metal stiffner trocar” of pig-tail catheter of “Bonte” brand) was performed immediately under ultrasound guidance(as shown in Fig. 1A-D), and human umbilical cord mesenchymal stem cell injection (1 × 10^7^/mL, 10 mL/bag) was transfused after successful catheterization, and the process of infusion was not less than 30 minutes.

### Safety monitoring

Subjects were followed in the hospital for immediate adverse events related with cell infusion (eg, anaphylactic reaction, fever, and bleeding) for 24 hours.

### Follow-up

Liver biopsy was performed before and 24 weeks after MSCs transplantation, and Ishak grading and staging were performed according to pathological changes.

The following data were recorded for patients before and after MSCs transplantation in weeks 4, 12, and 24.

(1) Portal main internal diameter and portal blood flow velocity;
(2) Liver Young’s modulus based on 2-dimensional shear wave elastic imaging measurement;
(3) The following quantitative parameters of CEUS for portal vein, hepatic parenchyma and hepatic vein: peak intensity, peak time and mean transit time;
(4) The levels of alanine aminotransferase, aspartate aminotransferase, albumin, and total bilirubin;
(5) The indexes of serological fiber: hyaluronic acid, N-terminal peptide of type III procollagen, laminin and type IV collagen;
(6) Coagulation function: prothrombin time(PT), prothrombin activity, activated partial thromboplastin time, and plasma fibrinogen;

### Statistical analysis

Statistical software SPSS26 was used for data analysis, and all the collected data were tested for normality. The data conforming to normal distribution were represented by mean ± standard deviation, and the rank data were represented by median and interquart (25% and 75%). The comparison of data at different time points was performed by one-way repeated measurement ANOVA. If *P* < .05, the comparison before and after treatment was performed. Wilcoxon labeled rank sum test was used to compare the changes in Ishak grading and staging scores of patients before and after stem cell transplantation. *P* < 0.05 was considered statistically significant.

## Result

### The “retentional metal stiffner trocar” of pig-tail catheter was successfully used to MSCs transplantation for LC

All 15 patients were successfully infused with MSCs via the portal vein with “retentional metal stiffner trocar” pig-tail catheter under the guidance of CEU (Fig. 1). Moreover, the drainage tube did not escape with respiration within 30 minutes after the successful insertion of MSCs without any external interference (such as abdominal band pressure). A small amount of perihepatic bleeding occurred in 1 patient (6.67%) during the operation. Pleural effusion developed in 2 patients (13.33%) and abdominal effusion was increased in 3 patients (20.00%) within 3 days after surgery. The other 9 patients did not have any other complications. In addition, all patients were followed up to week 24, and none was lost or died. These data indicated that the improved “retentional metal stiffner trocar” of pig-tail catheter was effective and safe in the MSCs transplantation.

### MSCs transplantation can alleviate some adverse changes associated with LC

As shown in Table 1, the levels of ALT, AST, TIBL, and ALB did not change significantly before and after MSCs transplantation. In the function of blood clotting, PT was lower than before with the treatment of MSCs transplantation. Also, we examined the indicators of liver fibrosis, including HA, LN, IV-C, and PCIIINP, and the results showed that the levels of HA and IV-C were significantly reduced after MSCs transplantation. To be specific, among the clinical indicators, PT, HA, and IV-C were statistically significant before and after transplantation. PT was decreased in 12 of 15 patients, HA was decreased in 13 patients, and IV-C was decreased in 9 patients before and 24 weeks after transplantation.

**Table 1.** The changes of liver function, coagulation function, and liver fibrotic indicators before and after MSCs transplantation.

Serum indicators	Before MSCsT	Week 4 after MSCsT	Week 12 after MSCsT	Week 24 after MSCsT	*F*	*P*
**Liver function**						
ALT (U/L)	28.00 ± 12.28	29.93 ± 10.71	27.80 ± 6.89	28.07 ± 9.11	0.301	.824
AST (U/L)	32.47 ± 8.12	31.53 ± 10.53	31.8 ± 8.69	31.8 ± 8.81	0.088	.966
TBIL (umol/L)	23.35 ± 13.06	19.30 ± 9.41	16.40 ± 9.94	19.55 ± 15.49	2.133	.149
ALB (g/L)	45.83 ± 4.32	44.26 ± 5.37	45.34 ± 4.59	46.39 ± 5.80	1.474	.235
**Coagulation function**						
PT(s)	13.02 ± 1.45	12.73 ± 0.93	12.67 ± 0.99*	12.53 ± 1.13*	3.22	.03
PTA(%)	75.83 ± 12.06	78.35 ± 10.86	79.30 ± 9.29	67.22 ± 21.41	2.07	.16
APTT(s)	26.33 ± 2.57	27.30 ± 2.06	27.11 ± 1.27	25.98 ± 7.39	1.64	.23
FIB (g/L)	2.28 ± 0.35	2.31 ± 0.53	2.27 ± 0.29	2.31 ± 0.35	0.06	.98
**Liver fibrotic indicators**						
HA (ng/mL)	295.84 ± 95.09	286.67 ± 91.76	285.85 ± 80.82	259.17 ± 85.16	3.59	.04
LN (ng/mL)	154.38 ± 46.3	154.89 ± 40.28	150.59 ± 54.36	149.44 ± 45.97	0.31	.82
IV-C (ng/mL)	148.59 ± 22.22	149.85 ± 27.69	135.86 ± 29.57	130.94 ± 35.80	2.90	.04
PCIIINP (ng/mL)	21.44 ± 7.02	21.41 ± 6.84	19.57 ± 7.21	21.44 ± 7.54	1.12	.35

Data are expressed as mean ± SD.

Abbreviations: ALB, albumin; ALT, alanine aminotransferase; APTT, activated partial thromboplastin time; AST, aspartate aminotransferase; FIB, plasma fibrinogen; HA, hyaluronic acid; IV-C, Type IV collagen; LN, Laminin; MSCsT, mesenchymal stem cells transplantation; N-terminal propeptide PCIIINP, procollagen III, PT, prothrombin time; PTA(%), plasma thromboplastin antecedent; TBIL, total bilirubin

**Figure 2.**
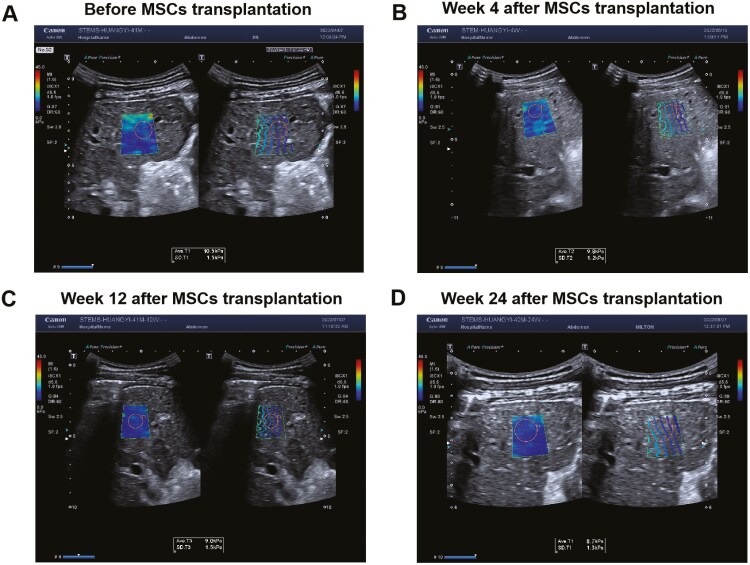
The ultrasonic images of liver Young’s modulus detected by TSWE before and after MSCs transplantation. A–D represents the changes of Young’s modulus before and after transplantation. Abbreviations: Ave: the average of Young’s modulus value. SD: standard deviation.

**Figure 3.**
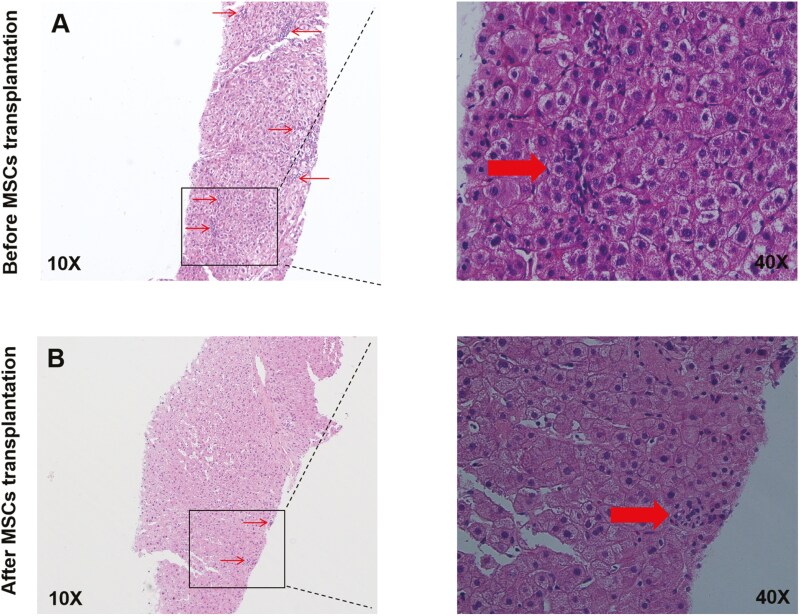
Pathological changes of liver before and after MSCs transplantation. HE staining was performed after liver biopsy in the same patient before and after stem cell transplantation. The arrows indicate the inflammatory necrotic cells. By the Ishak scoring system, A and B scored 3 and 2 under 10× lens, respectively.

Moreover, Young’s modulus was detected by TSWE, which was one of the indicators related to tissue stiffness, and it was decreased after MSCs transplantation in LC (Table 2 and Fig. 2). In addition, Ishak grading and staging scores and liver biopsy were used to assess cell death, the data showed that the Lytic necrosis was decreased after MSCs transplantation (Table 3 and Fig. 3). The above data suggested that MSCs transplantation can alleviate the liver damage caused by LC.

**Table 2.** The changes of liver Young’s modulus detected by TSWE before and after MSCs transplantation.

Time	Liver Young’s modulus (KPa)
Before MSCsT	10.36 ± 1.52
Week 4 after MSCsT	10.05 ± 1.49
Week 12 after MSCsT	10.19 ± 1.57
Week 24 after MSCsT	9.69 ± 1.21
F	3.23
P	0.03

**Table 3.** Changes of Ishak grading and staging scores before and after MSCs transplantation.

Time	Clastic necrosis	Fusion necrosis	Lytic necrosis	Portal inflammation	Score of fibrosis stage
Before MSCsT	2.00(1.00,3.00)	0.00(0.00,0.00)	2.00(1.00,2.00)	2.00(1.00,2.00)	5.00(5.00,6.00)
Week 24 after MSCsT	2.00(2.00,3.00)	0.00(0.00,0.00)	1.00(1.00,2.00)	2.00(1.00,2.00)	2.00(4.00,5.00)
Z	0.45	0.82	2.11	1.00	1.40
P	0.66	0.41	0.04	0.32	0.16

### MSCs transplantation had no significant effect on liver hemodynamics detected by CEU in LC

To evaluate whether MSCs transplantation has an effect on liver hemodynamics, the hemodynamic measurements were measured by portal vein diameter, blood flow velocity, portal venography parameters, hepatic parenchymal contrast parameters, and hepatic venography parameters.

As we can see, the portal vein diameter was decreased after MSCs transplantation. However, other parameters showed no significantly change (Table 4). These data suggested that MSCs transplantation had no significant effect on liver hemodynamics detected by CEU at 24 weeks follow-up.

**Table 4.** Liver hemodynamics detected by CEU in LC before and after MSCs transplantation.

Liver hemodynamics	Before MSCsT	Week 4 after MSCsT	Week 12 after MSCsT	Week 24 after MSCsT	F	*P*
**Portal vein diameter (cm)**	1.13 ± 0.22	1.16 ± 0.17	1.11 ± 0.21	1.04 ± 0.21	3.14	.04
**Portal blood flow velocity (cm/s)**	15.90 ± 5.89	16.98 ± 4.59	15.54 ± 3.97	16.98 ± 4.86	0.67	.57
**portal venography parameters**						
** PI(AU)**	50.05 ± 16.84	51.17 ± 17.2	51.45 ± 16.99	50.65 ± 16.97	2.44	.12
** TTP(S)**	24.75 ± 5.28	25.08 ± 5.08	25.45 ± 5.43	24.51 ± 5.36	1.32	.31
** MTT(S)**	44.65 ± 11.6	44.98 ± 12.12	45.38 ± 12.03	45.49 ± 11.5	1.71	.22
**Hepatic parenchymal contrast parameters**						
** PI(AU)**	47.31 ± 17.78	47.17 ± 17.06	47.09 ± 16.47	46.83 ± 18.17	0.32	.81
** TTP(S)**	29.50 ± 5.07	28.81 ± 7.21	31.70 ± 4.10	29.27 ± 7.21	1.05	.38
** MTT(S)**	46.73 ± 13.37	47.62 ± 12.98	47.20 ± 12.77	47.43 ± 13.56	0.60	.62
**Hepatic venography parameters**						
** PI(AU)**	49.38 ± 18.59	50.76 ± 17.78	49.46 ± 19.58	49.45 ± 18.15	1.58	.25
** TTP(S)**	43.52 ± 14.24	43.96 ± 14.32	44.21 ± 14.09	43.9 ± 13.68	0.87	.48
** MTT(S)**	61.41 ± 20.16	61.98 ± 20.10	61.63 ± 19.75	61.35 ± 19.83	1.08	.39

Data are expressed as mean ± SD.

Abbreviations: MTT, mean transmit time; PI, pulsation index; TTP, time to peak.

## Discussion

Our study showed that MSCs transplantation can alleviate some adverse changes of LC, consistent with other studies,^32-34^ so it is worth to further research. MSCs transplantation via the portal vein is safe and effective, but due to the hardness and thickened Glission sheath of LC, and the traditional catheter is soft, it is difficult to implant MSCs into the liver. In order to overcome this difficulty, MSCs transplantation was performed by using the “retentional metal stiffner trocar” of pig-tail catheter in this study. The “retentional metal stiffner trocar” of pig-tail catheter preserves the hard casing of stainless steel, resulting in a higher hardness for the outer sheath hose of pig-tail, which can enter the portal vein via the hard liver tissue and thickened Glission sheath. Moreover, it has directional control when entering the tube. Our results showed that this improved method is safe and effective, and is worthy of further promotion in clinical practice.

However, it is lack of a more comprehensive evaluation system to evaluate whether MSCs transplantation is effective in the treatment of LC. Our results showed that MSCs transplantation improves some traditional indicators, such as coagulation function and serum markers of fibrosis. However, the liver function was not significant improved, consistent with other study.^35,36^ Oppositely, some studies showed that MSCs transplantation can improve liver function in patients with LC.^37,38^ These conflicting results suggested that more clinical studies are needed to confirm whether MSCs transplantation can improve liver function in LC.

In addition to those traditional indicators for evaluating LC, our study performed a relatively comprehensive evaluation for LC by TSWE, CEU, and pathological changes. TSWE has been widely used to evaluate liver fibrosis and has a good diagnostic accuracy for LC.^39^ In this study, we detected a decreased level in Young’s modulus by TSWE after MSCs transplantation, one of the indicators related to LC. On the other hand, the hemodynamic changes are one of the clinical features of LC.^40,41^ The increased portal vein diameter is a risk factor for portal vein thrombosis in LC patients.^42^ Our study showed that the portal vein diameter was decreased after MSCs transplantation, which suggested that MSCs transplantation may reduce the risk of portal vein thrombosis. However, other data showed that MSCs transplantation had no significant effect on other liver hemodynamics detected by CEU at 24 weeks follow-up. In addition, we also compared the changes of liver histopathology before and after MSCs transplantation. The pathology of LC patients confirmed that MSCs transplantation can improve inflammation and cell necrosis in liver tissue. Our results confirmed that MSCs transplantation can improve some clinical indicators in patients with LC, so it is worthwhile to further explore the molecular mechanisms.

Although the basic research was not performed in this study, with the increase of basic research on MSCs therapy for LC, the molecular mechanism of MSCs-based treatment is gradually understood. Hepatic stellate cell (HSC) activation is the main source of excessive accumulation of collagen and extracellular matrix (ECM), leading to the occurrence of LC.^43^ Therefore, inhibiting the activation of HSC is very important in inhibiting the progression of LC, and MSCs can inhibit the activation of HSC by regulating the inflammatory microenvironment.^44^ In addition, other mechanism of MSCs inhibits liver fibrosis is as follows^45^: (1) MSCs participate in liver tissue regeneration and repair by differentiating into hepatocytes or fusing with hepatocytes. (2) MSCs can produce a variety of cytokines and growth factors, which may enable the regeneration of endogenous cells in damaged tissues via their ability to exert paracrine effects. (3) MSCs participate in immunomodulatory effects in liver diseases by regulating a variety of immune cells, such as natural killer cells, B lymphocytes, and T lymphocytes. However, the anti-fibrotic effects of MSCs is due to the exogenous MSCs after transplantation, and the long-term fibrotic microenvironment in LC can change the characteristics and function of MSCs,^16^ so the therapeutic effect of MSCS may be weakened with the extension of time. Thus, on the basis of previous studies, we will continue to explore the molecular mechanism of MSCs in the inhibition of liver fibrosis in the following study.

In summary, we successfully implanted MSCs into the portal vein by using the improved “retentional metal stiffner trocar” of pig-tail catheter, and evaluated the efficacy of MSCs therapy by a relatively comprehensive evaluation system. The data showed that the improved method was effective and safe, and the results showed that MSCs transplantation can improve some adverse indicators related to LC. However, there are some shortcomings as follows: (1)The number of patients in our study is small; (2) the follow-up time is not long enough; (3) lack of in-depth molecular mechanism investigation. Based on the above shortcomings, we will increase the number of patients and extend the follow-up time in subsequent studies. In addition, we will study the molecular mechanism of MSCs transplantation in the treatment of LC on the follow-up research.

## Conclusion

By a relatively comprehensive evaluation, MSCs transplantation can alleviate liver damage caused by LC. The improved “retentional metal stiffner trocar” of pig-tail catheter was effective and safe in the infusion of MSCs transplantation, which is worth promoting in the clinical practice.

## Supplementary material

Supplementary material is available at *Stem Cells Translational Medicine* online.

szae081_suppl_Supplementary_Material

## Data Availability

All data generated or analyzed during this study are included in this published article. The original data can be obtained from the Corresponding author.

## References

[1] Jeng WJ, Papatheodoridis GV, Lok A. Hepatitis B. Lancet. 2023;401:1039-1052. 36774930 10.1016/S0140-6736(22)01468-4

[2] Zhou WC, Zhang QB, Qiao L. Pathogenesis of liver cirrhosis. World J Gastroenterol. 2014;20:7312-7324. 24966602 10.3748/wjg.v20.i23.7312 PMC4064077

[3] Kramvis A, Chang K-M, Dandri M, et al. A roadmap for serum biomarkers for hepatitis B virus: current status and future outlook. Nat Rev Gastroenterol Hepatol. 2022;19:727-745. 10.1038/s41575-022-00649-z 35859026 PMC9298709

[4] Hsu YC, Huang DQ, Nguyen MH. Global burden of hepatitis B virus: current status, missed opportunities and a call for action. Nat Rev Gastroenterol Hepatol. 2023;20:524-537. 10.1038/s41575-023-00760-9 37024566

[5] Parola M, Pinzani M. Liver fibrosis: pathophysiology, pathogenetic targets and clinical issues. Mol Aspects Med. 2019;65:37-55. 10.1016/j.mam.2018.09.002 30213667

[6] Weiskirchen R. Hepatoprotective and anti-fibrotic agents: it’s time to take the next step. Front Pharmacol. 2015;6:303. 10.3389/fphar.2015.00303 26779021 PMC4703795

[7] Neuberger J. Liver transplantation in the United Kingdom. Liver Transpl. 2016;22:1129-1135. 10.1002/lt.24462 27081833

[8] Albillos A, Lario M, Alvarez-Mon M. Cirrhosis-associated immune dysfunction: distinctive features and clinical relevance. J Hepatol. 2014;61:1385-1396. 10.1016/j.jhep.2014.08.010 25135860

[9] Fu X, Liu G, Halim A, et al. Mesenchymal stem cell migration and tissue repair. Cells. 2019;8:784. 10.3390/cells8080784 31357692 PMC6721499

[10] Caplan AI. Why are MSCs therapeutic? New data: new insight. J Pathol. 2009;217:318-324. 10.1002/path.2469 19023885 PMC8793150

[11] Cao Y, Ji C, Lu L. Mesenchymal stem cell therapy for liver fibrosis/cirrhosis. Ann Transl Med. 2020;8:562. 10.21037/atm.2020.02.119 32775363 PMC7347778

[12] Huai Q, Zhu C, Zhang X, et al. Mesenchymal stromal/stem cells and their extracellular vesicles in liver diseases: insights on their immunomodulatory roles and clinical applications. Cell Biosci. 2023;13:162. 10.1186/s13578-023-01122-3 37670393 PMC10478279

[13] Squillaro T, Peluso G, Galderisi U. Clinical trials with mesenchymal stem cells: an update. Cell Transplant. 2016;25:829-848. 10.3727/096368915X689622 26423725

[14] Shi M, Li Y-Y, Xu R-N, et al. Mesenchymal stem cell therapy in decompensated liver cirrhosis: a long-term follow-up analysis of the randomized controlled clinical trial. Hepatol Int. 2021;15:1431-1441. 10.1007/s12072-021-10199-2 34843069 PMC8651584

[15] de Miguel MP, Prieto I, Moratilla A, Arias J, Aller MA. Mesenchymal stem cells for liver regeneration in liver failure: from experimental models to clinical trials. Stem Cells Int. 2019;2019:1-12. 10.1155/2019/3945672 PMC6525815 31191671

[16] Yang X, Li Q, Liu W, et al. Mesenchymal stromal cells in hepatic fibrosis/cirrhosis: from pathogenesis to treatment. Cell Mol Immunol. 2023;20:583-599. 10.1038/s41423-023-00983-5 36823236 PMC10229624

[17] Zhang Y, Li Y, Zhang L, Li J, Zhu C. Mesenchymal stem cells: potential application for the treatment of hepatic cirrhosis. Stem Cell Res Ther. 2018;9:59. 29523186 10.1186/s13287-018-0814-4 PMC5845383

[18] Barbash IM, Chouraqui P, Baron J, et al. Systemic delivery of bone marrow-derived mesenchymal stem cells to the infarcted myocardium: feasibility, cell migration, and body distribution. Circulation. 2003;108:863-868. 10.1161/01.cir.0000084828.50310.6a 12900340

[19] Kurtz A. Mesenchymal stem cell delivery routes and fate. Int J Stem Cells. 2008;1:1-7. 10.15283/ijsc.2008.1.1.1 24855503 PMC4021770

[20] Zhong Y, Tang Z, Xu R, et al. Effect of transplantation route on stem cell migration to fibrotic liver of rats via cellular magnetic resonance imaging. Cytotherapy. 2013;15:1266-1274. 10.1016/j.jcyt.2013.05.023 23993301

[21] Wang Y, Lian F, Li J, et al. Adipose derived mesenchymal stem cells transplantation via portal vein improves microcirculation and ameliorates liver fibrosis induced by CCl4 in rats. J Transl Med. 2012;10:133. 10.1186/1479-5876-10-133 22735033 PMC3439354

[22] Ramadori G, Saile B. Portal tract fibrogenesis in the liver. Lab Invest. 2004;84:153-159. 10.1038/labinvest.3700030 14688800

[23] Xu C, Hong Y. Rational design of biodegradable thermoplastic polyurethanes for tissue repair. Bioact Mater. 2022;15:250-271. 10.1016/j.bioactmat.2021.11.029 35386346 PMC8940769

[24] Lee SM, Ha H, Lee I, et al. Comparison between two-dimensional and point shear wave elastography techniques in evaluating liver fibrosis using histological staging as the reference standard: a prospective pilot study. Diagnostics (Basel). 2023;13:1646. 10.3390/diagnostics13091646 37175039 PMC10178401

[25] Kovatsch A, Honcharova-Biletska H, Segna D, et al. Performance of two-dimensional shear wave elastography and transient elastography compared to liver biopsy for staging of liver fibrosis. Eur J Clin Invest. 2023;53:e13980. 10.1111/eci.13980 36880934

[26] Wang P, Hu X, Xie F. Predictive value of liver and spleen stiffness measurement based on two-dimensional shear wave elastography for the portal vein pressure in patients with compensatory viral cirrhosis. PeerJ. 2023;11:e15956. 10.7717/peerj.15956 37727690 PMC10506585

[27] Chang GY, Fetzer DT, Porembka MR. Contrast-enhanced intraoperative ultrasound of the liver. Surg Oncol Clin N Am. 2022;31:707-719. 10.1016/j.soc.2022.06.007 36243503

[28] Mucke VT, Fitting D, Dultz G, et al. Application of contrast-enhanced ultrasound to detect hepatic hydrothorax in patients with liver cirrhosis. Ultraschall Med. 2022;43:473-478. 10.1055/a-1189-2937 32674185

[29] Liu Z, Li W, Zhu Z, et al. A deep learning model with data integration of ultrasound contrast-enhanced micro-flow cines, B-mode images, and clinical parameters for diagnosing significant liver fibrosis in patients with chronic hepatitis B. Eur Radiol. 2023;33:5871-5881. 10.1007/s00330-023-09436-z 36735040

[30] Dietrich CF, Bamber J, Berzigotti A, et al. EFSUMB guidelines and recommendations on the clinical use of liver ultrasound elastography, update 2017 (Long Version). Ultraschall Med. 2017;38:e48-e48. 10.1055/a-0641-0076 30176678

[31] Krishna M. Histological grading and staging of chronic hepatitis. Clin Liver Dis (Hoboken). 2021;17:222-226. 10.1002/cld.1014 33968379 PMC8087941

[32] Zhang Z, Lin H, Shi M, et al. Human umbilical cord mesenchymal stem cells improve liver function and ascites in decompensated liver cirrhosis patients. J Gastroenterol Hepatol. 2012;27:112-120. 10.1111/j.1440-1746.2011.07024.x 22320928

[33] El-Ansary M, Abdel-Aziz I, Mogawer S, et al. Phase II trial: undifferentiated versus differentiated autologous mesenchymal stem cells transplantation in Egyptian patients with HCV induced liver cirrhosis. Stem Cell Rev Rep. 2012;8:972-981. 10.1007/s12015-011-9322-y 21989829

[34] Xu L, Gong Y, Wang B, et al. Randomized trial of autologous bone marrow mesenchymal stem cells transplantation for hepatitis B virus cirrhosis: regulation of Treg/Th17 cells. J Gastroenterol Hepatol. 2014;29:1620-1628. 10.1111/jgh.12653 24942592

[35] Arbab AS, Thiffault C, Navia B, et al. Tracking of In-111-labeled human umbilical tissue-derived cells (hUTC) in a rat model of cerebral ischemia using SPECT imaging. BMC Med Imaging. 2012;12:33. 10.1186/1471-2342-12-33 23217090 PMC3538050

[36] Mohamadnejad M, Alimoghaddam K, Bagheri M, et al. Randomized placebo-controlled trial of mesenchymal stem cell transplantation in decompensated cirrhosis. Liver Int. 2013;33:1490-1496. 10.1111/liv.12228 23763455

[37] Kharaziha P, Hellström PM, Noorinayer B, et al. Improvement of liver function in liver cirrhosis patients after autologous mesenchymal stem cell injection: a phase I-II clinical trial. Eur J Gastroenterol Hepatol. 2009;21:1199-1205. 10.1097/MEG.0b013e32832a1f6c 19455046

[38] Mohamadnejad M, et al. Phase 1 trial of autologous bone marrow mesenchymal stem cell transplantation in patients with decompensated liver cirrhosis. Arch Iran Med. 2007;10:459-466. 17903050

[39] Leung VY, Shen J, Wong VW, et al. Quantitative elastography of liver fibrosis and spleen stiffness in chronic hepatitis B carriers: comparison of shear-wave elastography and transient elastography with liver biopsy correlation. Radiology. 2013;269:910-918. 10.1148/radiol.13130128 23912619

[40] La Villa G, Gentilini P. Hemodynamic alterations in liver cirrhosis. Mol Aspects Med. 2008;29:112-118. 10.1016/j.mam.2007.09.010 18177931

[41] D’Amico G, Morabito A, D'Amico M, et al. Clinical states of cirrhosis and competing risks. J Hepatol. 2018;68:563-576. 10.1016/j.jhep.2017.10.020 29111320

[42] Dong G, Huang X-Q, Zhu Y-L, et al. Increased portal vein diameter is predictive of portal vein thrombosis development in patients with liver cirrhosis. Ann Transl Med. 2021;9:289. 10.21037/atm-20-4912 33708916 PMC7944309

[43] Bataller R, Brenner DA. Liver fibrosis. J Clin Invest. 2005;115:209-218. 10.1172/JCI24282 15690074 PMC546435

[44] Meier RP, Mahou R, Morel P, et al. Microencapsulated human mesenchymal stem cells decrease liver fibrosis in mice. J Hepatol. 2015;62:634-641. 10.1016/j.jhep.2014.10.030 25450712

[45] Yao L, Hu X, Dai K, et al. Mesenchymal stromal cells: promising treatment for liver cirrhosis. Stem Cell Res Ther. 2022;13:308. 10.1186/s13287-022-03001-z 35841079 PMC9284869

