# Supplementary material for: A comprehensive evaluation system for ultrasound-guided infusion of human umbilical cord-derived MSCs in liver cirrhosis patients
Source: Stem Cells Transl Med. 2024 Nov 8;14(1):szae081. doi: 10.1093/stcltm/szae081 (PMC11821905; doi:10.1093/stcltm/szae081)

1. Table 1. Clinical data of patients with LC

Patients	Male(n)	Female(n)	Age (year)	Disease course (year)	BMI (kg/m^2)	Child-Pugh B (n)
Clinical data	13	2	47.73±7.08	20.33±9.75	23.79±2.57	15

2. Fig.1 The flowchart of the whole clinical trial

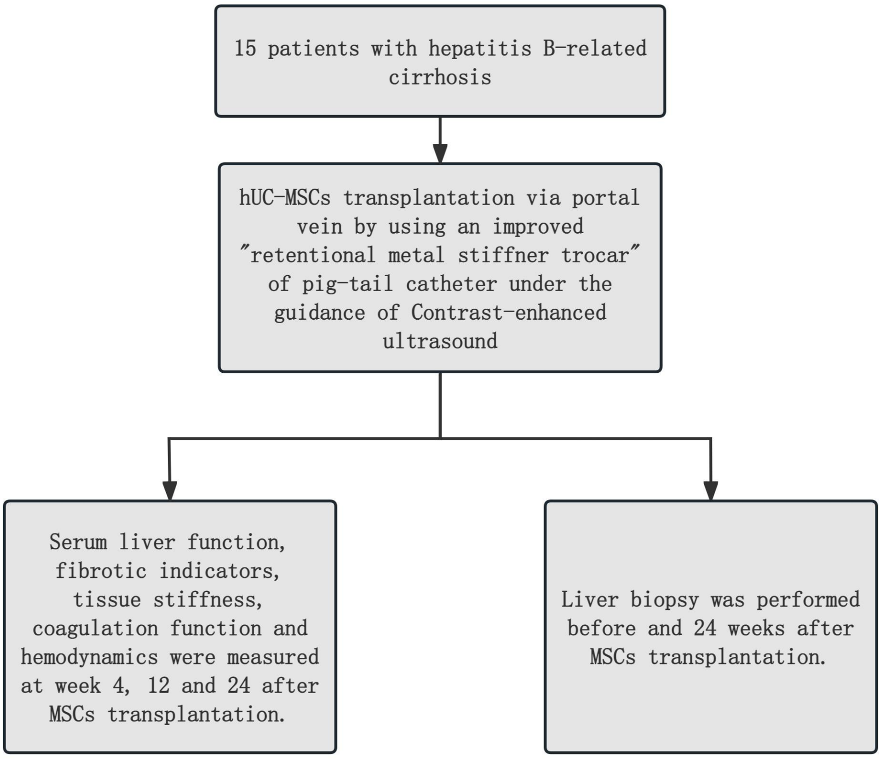

Supplement: szae081_suppl_Supplementary_Material [file szae081_suppl_supplementary_material.pdf]
